# Insidious Onset of Pulmonary Langerhans Cell Histiocytosis During Oncological Follow-Up

**DOI:** 10.5334/jbsr.2913

**Published:** 2022-10-20

**Authors:** Nico Hustings, Eveline Slachmuylders, Adriana Dubbeldam

**Affiliations:** 1 UZ Leuven, BE

**Keywords:** Pulmonary Langerhans Cell Histiocytosis, High-Resolution CT, Anatomopathology, Lung, MAPK

## Abstract

**Teaching Point:** Pulmonary Langerhans cell histiocytosis (PLCH) has to be included in the differential diagnosis of cystic pulmonary lesions on chest computed tomography (CT). CT has important diagnostic value by demonstrating initial centrilobular nodules that in time cavitate and transform into cysts, typically sparing the costophrenic angles.

## Case History

**Figure 1:**
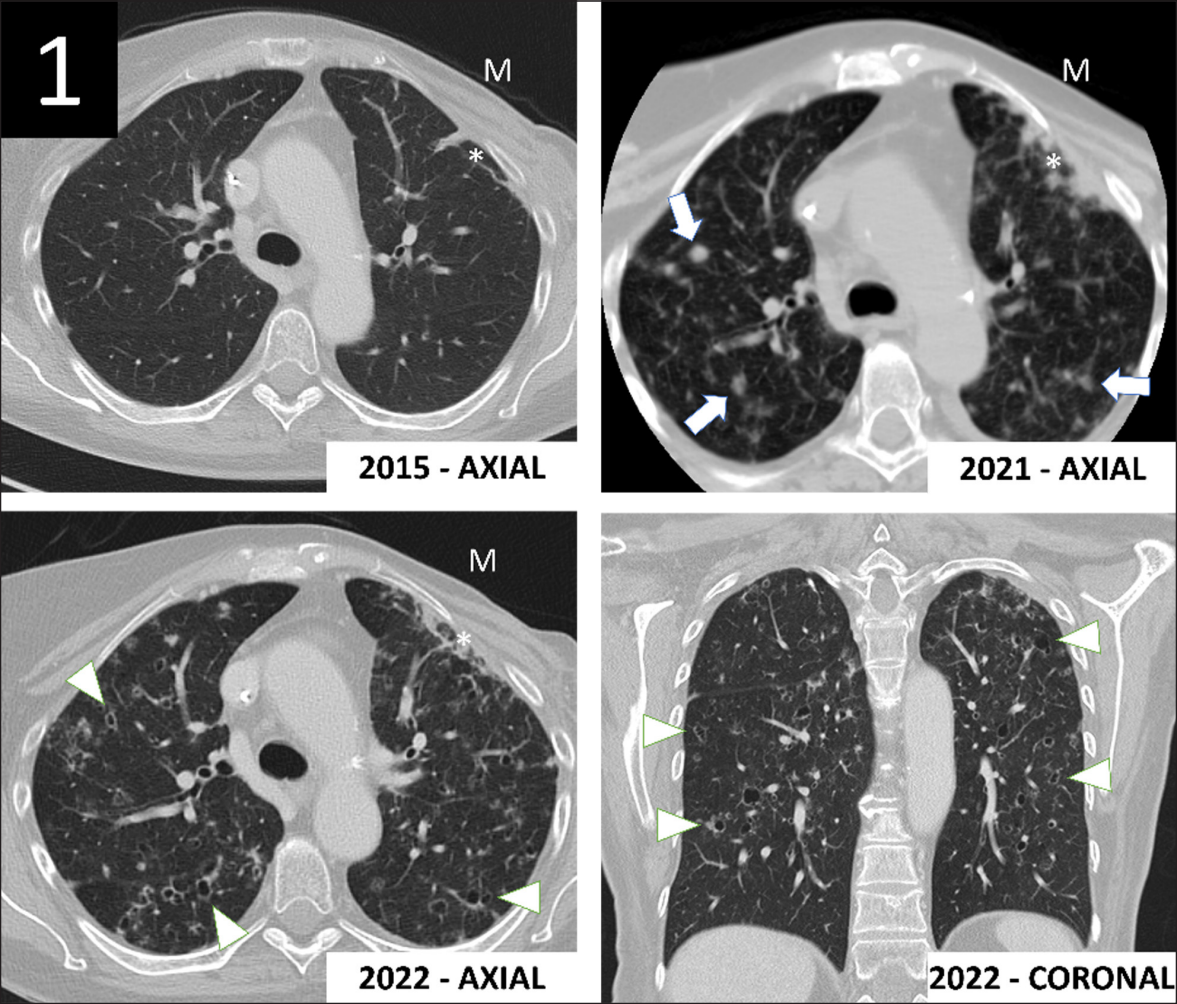


A 65-year-old woman was in follow up for a curatively treated breast cancer in 2014. Medical history mentioned heavy smoking behavior of 35 pack years (Figure 1). A surveillance CT scan in 2015 showed left-sided status after mastectomy (M) with focal pulmonary radiofibrosis (*) and no focal pulmonary lesion. A CT scan in 2021 showed disseminated centrilobular nodules (white arrows). A follow-up CT in 2022 revealed cystic transformation of these previously solid lesions (arrowheads). The coronal plane shows the distribution of these cysts in the upper and middle parts of the lungs, sparing the costophrenic angles. At this time, the patient had elevated tumor marker CA 15–3 and inflammatory markers, without physical complaints. Clinically and radiologically, the differential diagnosis consisted of cystic lung metastases versus pulmonary Langerhans cell histiocytosis (PLCH).

**Figure 2:**
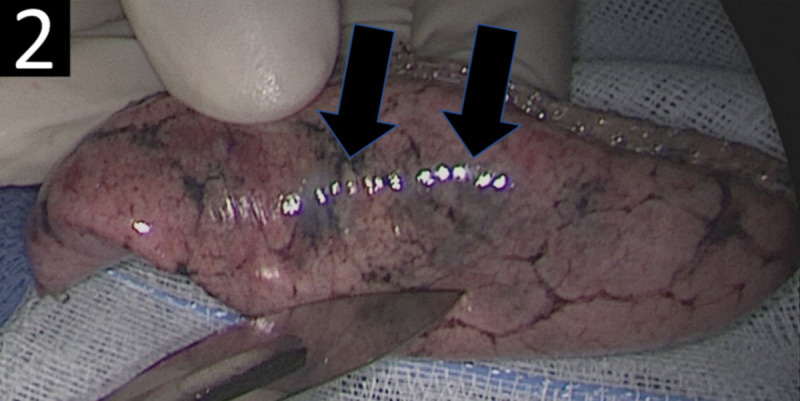


A lung biopsy of the right upper lobe was performed. Visual inspection of the specimen showed multiple bluish colored nodular lesions (Figure 2, black arrows).

**Figure 3:**
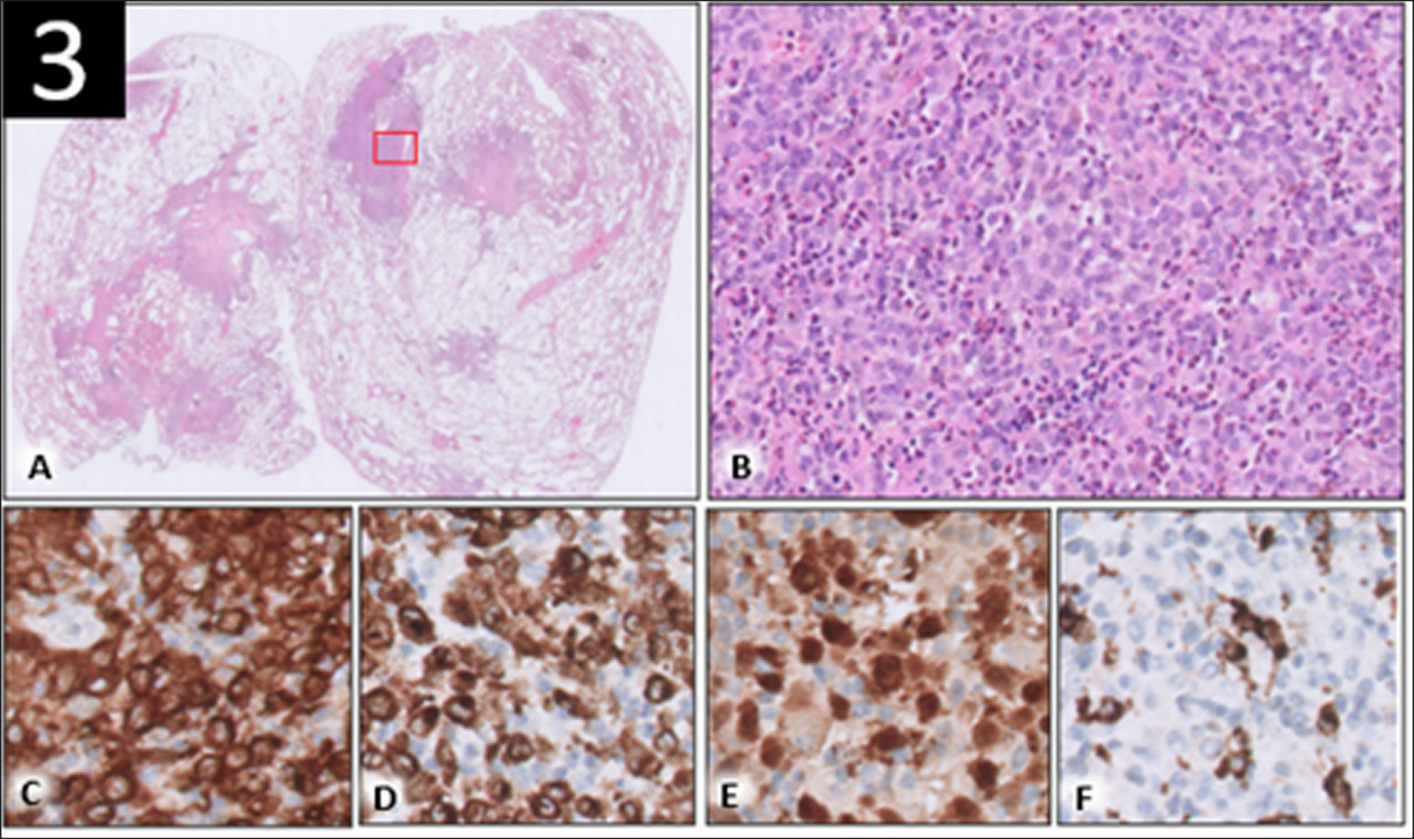


Histopathological examination revealed lung parenchyma with scattered stellate nodules. Some nodules showed central scarring, and some were associated with small cystic spaces (Figure 3A). The nodules consisted of aggregates of Langerhans cells, numerous eosinophils and a variable amount of monocytes and lymphocytes (Figure 3B). The Langerhans cells contained convoluted nuclei and pale, mildly eosinophilic cytoplasm. Langerhans cells were positive for CD1a, S100 and langerin (Figure 3C–E). CD68 was positive in the scattered histiocytes but negative in the Langerhans cells (Figure 3F).

The patient was urged to stop smoking. Depending on the evolution of the lung lesions, additional therapy had to be considered, that is, chemotherapy or targeted therapy with a BRAF-kinase inhibitor.

## Comment

PLCH is a rare (estimated prevalence of 1–2/1,000,000) neoplastic disorder consisting of bone marrow-derived Langerhans cells infiltrating the lungs, accompanied with a strong inflammatory response [1]. It is caused by an activation of the mitogen-activated protein kinase/extracellular signal-regulated kinase signaling pathway. PLCH typically occurs in young smokers, with mostly non-specific clinical presentation (cough, dyspnea, chest pain, fever) and in 5–25% of cases without any clear symptoms. Conventional chest radiography has limited value, unless in patients with advanced disease. Chest CT is important for diagnosis by demonstrating centrilobular nodules which transform into cysts that may convolute, and typically sparing the costophrenic angles. Histopathology shows characteristic eosinophilic granulomas with the presence of Langerhans cells that display antigen CD1a. Treatment of PLCH consists of smoking cessation and various chemotherapy regimens. Some patients qualify for targeted therapy if tested positive for mutations in the genes responsible for the malfunctioning signaling pathway. Prognosis of PLCH is unpredictable and variable [1].
